# When TV neighbours become good friends: Understanding *Neighbours* fans’ feelings of grief and loss at the end of the series

**DOI:** 10.1371/journal.pone.0302160

**Published:** 2024-06-12

**Authors:** Adam Gerace

**Affiliations:** 1 College of Psychology, School of Health, Medical and Applied Sciences, CQUniversity, Adelaide, South Australia, Australia; 2 Appleton Institute, CQUniversity, Adelaide, South Australia, Australia; Shandong University, CHINA

## Abstract

Fans may experience significant upset and distress when a television series ends. However, grief and loss reactions to the end of a fictional series have seldom been investigated. It is likely that the degree to which such reactions are felt is influenced by viewing motives (e.g., pleasure, meaning making), connection to the series and its fan community, relationships formed with characters, including parasocial bonds and experiences of empathy, and tendency to engage with others’ perspectives and emotions, including fictional characters. The purpose of this study was to examine predictors of fans’ grief and loss reactions to the end of the television series *Neighbours*, which aired from 1985 to 2022. Fans (*n =* 1289) completed an online survey shortly after the screening of the final episode. The survey measured grief emotions and cognitions, acceptance that the series had ended, distress at the loss of a parasocial relationship with a favourite character, feelings of closure, and expressions of gratitude for the series. Predictors of these grief and loss reactions examined in the survey were viewing motives, fan identity, strength of a parasocial relationship formed with a favourite character, empathy towards that character, and tendency to take others’ perspectives, experience empathic concern and personal distress, and tendency towards engagement with fictional characters. Greater grief and loss reactions were experienced by fans whose motives for watching involved being entertained and exposed to different lifestyles, who felt a stronger fan connection to the series, and who formed stronger parasocial empathic relationships with their favourite character. Factors such as gender, age, and empathic tendencies predicted various types of grief reactions. Understanding fan experiences when a long-running series ends advances theory and research on viewer parasocial relationships and engagement with media, as well as providing evidence that the loss of a series or favourite character can be viewed as a type of grief experience.

## Introduction

In March 2022, Australian TV series *Neighbours*, which began screening in 1985 [1], was cancelled. The announcement followed speculation for some time regarding the 37-year-old show’s future, and years of audience decline in its home country for the once very popular show [2]. Despite this, the announcement was accompanied by considerable upset in Australia and particularly the UK, the latter where the series had maintained a loyal following and high ratings [3–7]. The implications for the Australian television industry (e.g., loss of employment for crew, training opportunities for emerging performers) of the end of a multiple-day-a-week series were discussed [8, 9]. From a viewer perspective, the media reported on fans’ experiences of sadness, anger, and in some cases, reactions referred to as ‘grief’ when faced with the end of the series [10, 11].

Celebration of the series and acknowledgement of its importance to fans was evident in the months leading up to the final episode [12]. The 90-minute finale of *Neighbours* screened 28 July in Australia and 29 July in the UK (with the finale screened in other countries later), and in both cases drew large audiences [13, 14]. The finale was praised by fans as a fitting close to the series and a farewell to both current and retuning characters [15]. The series’ theme song for its entire run ends with the refrain “that’s when good neighbours become good friends” [16], reflecting how understanding and being there for one another are key ingredients to fostering intimacy among those living in close suburbia. Based on fans’ reactions, it seemed audiences were saying goodbye to the fictional people of Ramsay Street who had, indeed, become *very* good friends.

Fan reactions to the conclusion of *Neighbours* suggested that they were processing both the end of the television series and their relationships with its characters. In doing so, they were demonstrating grief reactions similar to those involved in the end of real-life relationships [e.g., 17, 18], including negative emotions and cognitions, attempts at closure, and gratitude for what they had experienced through these relationships. Such reactions would benefit from a psychological consideration of the motives and connections formed with fictional worlds and their inhabitants.

The purpose of this study was to investigate what factors predicted greater grief and loss reactions from *Neighbours* fans in response to the end of the series. To address this question, it is important to examine viewers’ reasons for watching fictional media and the ways in which they experience relationships with fictional characters.

### Viewer motives, fan identity, and parasocial empathy relationships

Why might viewers experience grief or loss at the end of a series? Viewing motives need to be considered when examining connections with fictional media. Motives for engaging in fictional media might include varied reasons such as for excitement, for company, to be exposed to situations one has not experienced, for social interaction with fellow viewers, to pass the time, to stimulate self-reflection, and for emotional release [19–25]. Viewing motives have usefully been conceptualised as reflecting hedonic and eudaimonic needs [26, 27]. Hedonic motives involve the generation of positive, enjoyable, or pleasurable emotions, while eudaimonic motives involve using media for meaning-making, reflection, insight, and growth [20]. In considering the experiencing of emotion through fictional media, researchers have further suggested that gratification comes from not only pleasant affect, but also more intense emotions (e.g., fear, suspense) and even experiencing of sad emotions, which provide the opportunity for empathic or sympathetic involvement with characters [20, 23, 28, 29]. Such vicarious immersion in and witnessing of narrative situations can heighten viewers’ emotions, but these experiences remain safe for the viewer [30]. Within the eudaimonic meaning-making approach, viewers may seek out media that allows them to consider their identity and world views, as well as to enhance their social relationships through real-world relationships with other fans and the fandom [31–33]. An important aspect of this meaning-making is the extent to which one feels bonded with a series. Fan identity has been found to include personal aspects (e.g., passion for a series, considering oneself a ‘big’ fan), perceptions of impact on one’s life (e.g., extent to which the media has helped clarified one’s values and facilitated growth), and social interaction (e.g., communication with other fans) [34].

The benefits of meeting needs or gratification through media viewing have not been directly investigated as much as might be expected [35, 36]. However, both hedonic and eudaimonic entertainment experiences were found in one study [37] to be associated with feelings of increased vitality, with hedonic experiences providing the specific potential for relaxation and detachment, and eudaimonic experiences involving the potential for mastery or learning. More specific to fan identity, greater social interaction with fellow fans of a series or film is associated with greater satisfaction with personal and family relationships [34]. These findings suggest that to the extent viewers use a television series to meet important needs, they may be more grief-stricken and upset when that series ends.

A fundamental way in which individuals engage with fictional media is through characters. Research into fans’ engagement with media has found that they experience relationships with fictional characters and personalities. This phenomenon, originally termed a *parasocial interaction*, was described in the 1950s [38] within the context of in-the-moment ‘interactions’ with television performers, particularly on-air personalities, perhaps owing to the style of direct-to-camera presentation by hosts and the prevalence of variety and talk shows in television’s early years that invited such an identification. However, later work has found that this involvement “can extend beyond any single viewing episode” ([39], p. 21), with the potential for a *parasocial relationship* to develop. Parasocial relationships are described as “a one-sided interpersonal relationship that television viewers establish with media characters” ([40], p. 280).

There has been debate regarding the most appropriate way to measure the parasocial concept (e.g., interactions vs. relationships [39]), and there is difficulty in devising uniform measures given the need to tailor according to the context, such as measuring a relationship with a personality (e.g., host, newsreader, celebrity) or fictional character. Regardless, such relationships can be considered [41] to exist along the continuum of all possible social interactions, both real-world and media-mediated, from face-to-face real-world social encounters to varying types of parasocial encounter that involve the development of a relationship. Parasocial relationships do, however, differ from those in the real world, according to this view, since characters are not real and offer no chance of interaction or an actual relationship [41]. However, as with real-world social relationships, identification with these characters can occur, these one-sided relationships can evolve based on other encounters (e.g., discussing a character with fellow fans), and interaction can be simulated if not enacted.

Parasocial relationships involve feelings of attraction or liking, perceptions of similarity and familiarity to oneself, and expressions of empathy for characters [19, 29, 40, 42, 43]. The strength of the relationship and perceptions of intimacy or feelings of knowing the character are associated with some of the same psychological and personality characteristics that have been found to influence relationship behaviours in real-world scenarios, with attachment styles particularly investigated (e.g., [44–47]; for reviews of the wider research, see [48, 49]). Parasocial interaction and the development of a parasocial relationship are also associated with a range of responses to the recipient of the interaction. Greater parasocial interaction or relationships with media personalities/characters predict continued involvement (e.g., with the show), utilising the personality or character as source of information, changing attitudes and behaviours in line with the perspectives of the personality/character, and increased perceptions of belonging [50–53]. Like viewing motivates more generally, these relationships meet a range of needs, and it is likely that the end of such relationships may be felt more acutely depending on the strength of the parasocial bond.

An aspect particularly influential in explaining closeness or parasocial bonding with characters is empathy. Empathy is a multidimensional phenomenon consisting of cognitive and emotional processes and outcomes [54–58]. Empathy involves experiencing emotions in response to another person’s situation, including the same or similar emotions to that person (parallel feelings or emotional matching, e.g., sadness when that person is sad), as well as emotional responses to, rather than replication of that person’s emotion [56, 60]. These latter responses may include empathic concern or care for that person, as well as distress at their plight. Such reactive emotions often come about through cognitive empathic processes, with the ability or tendency to psychologically put oneself in the other person’s place considered the main or at least most cognitively complex of empathic responses to others’ experiences [54, 57, 58]. Empathy fosters social connection between people [53, 57], and so it stands to reason that perceptions and experiences of a closer parasocial relationship are influenced by empathy, and perceptions of closeness based on aspects such as perceived similarity [59–61] would in turn influence greater empathy towards characters.

Empathy toward characters across a range of mediums results in changes in viewers’ attitudes, perspectives, and emotions. Studies examining television, film, live theatre, and literature have found that narrative immersion and empathic connection with characters is associated with endorsement of behaviours consistent with the narrative message, changes in attitudes towards groups depicted in the narrative, and altruism towards these groups [53, 62–66].

Researchers have acknowledged that empathy is a part of the parasocial relationship [61], and both definitions and scales examining parasocial relationships and more general viewing motives often include reference to or items tapping empathic processes and outcomes (e.g., [19, 67]). Many aspects of empathic relationships are present within conceptualisations of the parasocial encounter, including perceptions of similarity and closeness (e.g., bonding, similar thinking), considering how one’s past experiences relate to what the character is going through, emotional matching, and vicarious emotional experience [19, 20, 68]. Indeed, empathy has been considered to underlie identification with a favourite character, with identification involving almost assuming the character’s perspective and thoughts and feelings entirely [69]. While identification or a blurring of the distinction between self and other is considered an aspect of empathic emotional matching [69], by contrast perspective taking and emotional reactions to another’s situation are seen to involve awareness of the self-other distinction [60, 69–71]. Interestingly, however, perceived cognitive identification with a favourite character was found in one study [72] to be associated with perspective taking and empathic concern, but not emotional matching or contagion, suggesting the complexity of these relationships [30, 73].

A few studies have examined the relationship between parasocial relationships with a favourite character and empathy towards that character. An early study [24] into this relationship with soap opera characters found that participants who felt they could predict the attitudes, feelings, and behaviours of their favourite character (a potential outcome of perspective taking) reported a stronger parasocial relationship with that character. Other studies have found that people with lower self-esteem who perceive a celebrity to be closer to their ideal self empathise to a greater degree with the celebrity’s positive and negative experiences [61]. Investigation with diverse types of parasocial figure, such as digital celebrities (e.g., bloggers), have found that empathy, defined as feeling parallel feelings to the celebrity and believing one can discern their underlying emotions, is associated with perceptions of a greater parasocial relationship with them [74]. A meta-analysis [49] found a strong association between greater character identification and a stronger parasocial bond. As an outcome, favourite characters, and those whom fans consider they know better and are like them, are perceived as more real [75], suggesting an entering into their fictional worlds and perspectives.

At a wider level, individuals differ in the extent to which they engage in perspective taking, experience emotional reactions to others’ experiences, and indeed the extent to which they are more prone to engage with fictional characters (termed fantasy) [53, 76–79]. In previous studies, greater tendency towards empathic concern [80], perspective taking [81], and emotional contagion (feeling what the other is feeling) [82] were associated with stronger parasocial relationships. In a study [83] that investigated the relationship between empathy and the strength and nature of participants’ parasocial relationships, stronger parasocial relationships with a favourite character were significantly associated in multiple regression analysis with participants’ greater tendencies to taking the perspectives of others (perspective taking) in everyday life and to empathise with fictional characters in various mediums (fantasy). Furthermore, greater tendency to perspective taking, fantasy, and experiencing of distress when exposed to others’ difficult and negative emotional experiences (personal distress) were significantly associated in multiple regression with greater commitment to the parasocial relationship; greater personal distress was also significantly related to greater feelings of having invested in the parasocial relationship. Empathic concern in this study was associated with both greater satisfaction with and commitment to a parasocial relationship in correlational analysis, but not when empathic concern was considered with other empathy measures in regression analyses. In a study [84] considering the relationship between empathic responsiveness in everyday life and empathic involvement with fictional characters, greater tendencies towards real life perspective taking, empathic concern, and personal distress were associated with greater tendencies to take the perspective of fictional characters and to experience empathic concern for them and personal distress when they are involved in negative experiences.

Overall, studies suggest that when a person empathises with a favourite character to a greater degree, or has a greater tendency towards empathy, they feel closer to and experience a stronger parasocial bond with a favourite character, even if the effects for different types of empathy are varied. Such empathic relationships are also important to shaping cognitions and attitudes (e.g., [53]). Therefore, the degree of empathy felt may be an important predictor of how individuals respond when these parasocial relationships end.

### Parasocial grief and loss at the end of a series

Like social or real-world relationships, parasocial relationships can end. The severing of ties with characters has been termed a *parasocial breakup* [44, 45, 85]. Within the context of a television series, this may be necessitated, for example, by the decision of an actor to leave, or the end of a series. A character’s end may be handled by their leaving the fictional location or, particularly in television dramas, through death. Fictional deaths in soap operas and serials have a long history, notably within Australia with the death of character Molly Jones on *A Country Practice* [86]. In such cases, fans experience grief-type distress and exhibit behaviours that may blur the boundaries between a fictional character’s demise and that of a real person [86, 87].

Grief and loss responses to the end of fictional relationships share commonality with grief responses at the end of real-world social relationships (e.g., [17, 18, 88]). Surprisingly, there has not been a lot of direct investigation into how relationship endings with characters and series are associated with experiences of grief. Most investigations have focused on examining existing data. Studies of deaths of popular fictional characters, such as in the television series *House* [89], *Game of Thrones* [90], *Grey’s Anatomy* [91], and *This Is Us* [92], for example, analysed social media posts related to characters’ deaths. Across studies, fans reported feelings (e.g., shock, sadness), cognitions (e.g., longing), behaviours (e.g., performing memorialising rituals), and trajectories that researchers found could be understood utilising concepts and models within real-world grief literature.

In a study [42] that examined predictors of parasocial breakup intensity at the end of a television series, students’ reactions to the end of the US television series *Friends* were investigated shortly after the final episode aired. To measure parasocial breakup intensity, participants reported on their feelings (e.g., sadness) and experiences (e.g., thinking about one’s favourite character) of the end of their relationship with their favourite character. Greater parasocial distress was predicted by greater commitment and affinity towards the show, a stronger parasocial relationship with one’s favourite character, and greater experiencing of loneliness. Other studies that have examined actual [93] or potential [45] series endings, or the real-life deaths of celebrities (e.g., Robin Williams; [94]) support the strength of the parasocial relationship as an important factor in anticipated or actual grief reactions. One study [95] that examined disruption to television viewing due to the 2007–2008 Writers Guild of America strike found that greater parasocial distress was experienced by participants who reported a stronger parasocial relationship with their favourite character, placed greater importance on television in their lives, and by those whose television viewing motivations were more instrumental, that is for motives such as entertainment, arousal, and social interaction.

### The present study

The conclusion of *Neighbours*, a long-running series, provided a rare and timely opportunity to examine fans’ grief experiences following the end of potentially very established parasocial relationships. It allowed for investigation of what factors predict greater or lesser fan grief and loss reactions. Several factors were investigated, namely motives for watching the series (e.g., for entertainment, to pass time, to vicariously experience new situations, to connect with other fans), self-identification as a fan and feeling part of the fandom, the parasocial relationship formed with a favourite character, empathy for this character, and tendency to engage in perspective taking, experience empathic concern and personal distress, and to engage with fictional characters (fantasy).

It was anticipated that greater grief and loss reactions to the end of *Neighbours* would be predicted by:

1. Greater motives for viewing the series for entertainment, experiencing of different situations and lifestyles, and feeling part of a fan community, and lesser motives for viewing the series to pass the time.
2. Greater self-identification of being a fan of the series and being part of the series fandom.
3. Greater parasocial interaction (parasocial relationship) with a favourite character.
4. Greater empathy for a favourite character.
5. Greater tendency to experience perspective taking, empathic concern, fantasy, and personal distress.

Based on work outside of the media engagement literature (e.g., [17, 18], an important part of a series ending may be some eventual form of closure and gratitude for experienced parasocial relationships. Investigation of degree of closure and gratitude were, therefore, also examined as indicators of grief reactions. Since fans participated in the study shortly after the final episode aired, meaning experiences of closure and gratitude might be more initial, hypotheses were more tentative. However, it was anticipated that lesser closure and greater gratitude would be predicted by greater engagement with the series, in the form of greater viewing for entertainment, experiences, feeling a part of the community, and lesser viewing to pass the time; greater self-identification as a fan and with the fandom; and greater parasocial interaction with and greater empathy towards a favourite character. Specific hypotheses about empathic tendencies were not formulated.

## Method

### Design

The study was a correlational design examining predictors of grief and loss in fans of *Neighbours* through an anonymous online survey.

### Participants

Participants were eligible to complete the survey if they (a) considered themselves a fan of the series; (b) had watched, on average, at least one episode a week of the series over the past 12 months; and (c) had watched the final episode of the series. Restricting participation to watching at least one episode a week (in recent years, the series aired 4 and 5 episodes a week in Australia and the UK, respectively) ensured that participants were somewhat active current watchers of the series, rather than, for example, former viewers who considered themselves once a fan of the series. Requiring viewing of the final episode was so that feelings and thoughts measured would be about the *actual* end of the series (and participants had experienced the loss), rather than experiences leading up to the anticipated end of the series.

Participants were recruited via social media posts to Facebook and Twitter groups. For Facebook, a recruitment text and URL for the survey were posted with permission of group administrators to two fan groups associated with the series: “*Neighbours*” [96] and “*Neighbours*: Past, Present, and Future” [97]. For Twitter, the recruitment text and URL were posted on the researcher’s Twitter page. Twitter users with public profiles who identified themselves as *Neighbours* fans and had a large number of followers were also messaged publicly and asked if they would retweet the researcher’s original tweet. Recruitment via social media and specific *Neighbours* groups meant that it was likely more devoted fans were recruited, but this was considered an appropriate strategy given the time imperative to recruit participants close to the final episode screening and the need to include engaged fans.

The final sample consisted of 1289 respondents (76.34% female) who completed the survey. Table 1 presents participant demographics and viewing habits. Mean age was 45.12 years (*SD =* 10.63, *Range =* 18–90 years). Most participants lived in England (68.04%) or Australia (13.42%). Participants were long-time viewers, with 512 (39.72%) respondents having watched the series for 37 years (the entire run). Over 90% of participants viewed an average of five episodes a week, with the majority of participants watching alone, and mostly through a television station or a combination of television and streaming.

**Table 1:** Participant demographics and viewing habits.

Variable	*N* (%)
Gender	
Female	984 (76.34)
Male	286 (22.19)
Non-binary or gender diverse	8 (0.62)
Prefer not to say	9 (0.70)
Other	2 (0.16)
Country	
England	877 (68.04)
Australia	173 (13.42)
Scotland	97 (7.53)
Wales	61 (4.73)
Republic of Ireland	25 (1.94)
Northern Ireland	23 (1.78)
Other	33 (2.56)
Years Watching	
1 year	0 (0.00)
2–6 years	12 (0.93)
7–11 years	31 (2.40)
12–16 years	43 (3.34)
17–21 years	89 (6.90)
22–26 years	110 (8.53)
27–31 years	156 (12.10)
32–36 years	336 (26.07)
37 years (entire run of series)	512 (39.72)
Average episodes watched per week	
1 episode	5 (0.39)
2 episodes	7 (0.54)
3 episodes	21 (1.63)
4 episodes	50 (3.88)
5 episodes	1181 (91.62)
Other	25 (1.94)
Viewing method	
Television (e.g., 10 Peach, Channel 5)	814 (63.15)
Streaming service (e.g., 10 Play, My5)	140 (10.86)
Combination of television and streaming	300 (23.27)
Other	35 (2.72)
Watched	
Mostly alone	660 (51.20)
Mostly with family, friends, or partner	291 (22.58)
Mix of alone and with others	324 (25.14)
Other	14 (1.09)

### Materials

Participants first completed a series of demographic items and items about viewing habits (e.g., years watching the series, method of viewing the series).

To measure study variables, existing, new, and adapted measures were used. Existing measures were used when available; in this case, existing measures of parasocial breakup (Parasocial Breakup measure; [42]) and empathic tendencies (Interpersonal Reactivity Index; [76, 77]) were utilised. New items were written to measure grief experiences, closure and gratitude, degree of being a fan, and empathy towards characters. This was deemed necessary as suitable measures were not found; for example, measures of real-life grief were not considered appropriate, and there was a paucity of investigation of empathy for characters that used a multidimensional approach to understanding empathy (e.g., consideration of both cognitive and emotional components).

Two measures of concepts investigated in the present study required adaptation. These were measures of viewing motives (Local Television News Viewing Motives scale; [19]) and parasocial relationships (Parasocial Interaction Scale; [19]). Both measures had been used in various forms in previous research, with researchers adapting them as needed (e.g., [42]), since they were originally developed to investigate news program viewing and relationships with newscasters. Items were rephrased to pertain to the *Neighbours* television series and characters, with additional items added to measure concepts considered important to a drama series such as exposure to different lifestyles (e.g., [27]) and to reflect a move within *Neighbours* towards reflecting more contemporary issues and diverse lifestyles in recent years (see [98–101]).

To determine the underlying factor structures of newly developed items to inform scale construction, and to investigate suitable factor structures of adapted measures, items were grouped into six sets of items: (1) grief and loss reaction items; (2) closure and gratitude items; (3) motives for watching items; (4) degree of being a fan items; (5) parasocial relationship items; and (6) empathy towards a favourite character items. For each set of items, the following procedure was undertaken based on guidelines [102, 103] for determining factor structure of items. First, parallel analysis [104], using a Monte Carlo simulation with 1,000 replications, was used to determine number of factors to specify in subsequent exploratory factor analysis. Following this, principal axis factoring (direct oblimin rotation) was undertaken, specifying the fixed number of factors based on the results of the parallel analysis. For individual items to be retained on a derived factor, they had to load at least .32 on the factor [102]. For parasocial relationship and empathy items, an additional analysis was undertaken, with all items included together in a final exploratory factor analysis. This was done based on the very high correlation between two separate scales and is explained in more detail below where derived scales are described. Parallel and factor analysis was not deemed necessary for the existing measures of parasocial breakups and empathic tendencies.

For all items in the survey except the Interpersonal Reactivity Index [76], participants utilised a five-point Likert-type response scale with the anchors 1 = *Strongly disagree*; 2 = *Disagree*; 3 = *Neither disagree nor agree*; 4 = *Agree*; and 5 = *Strongly agree* to answer individual items in scales. Respondents completed items in the Interpersonal Reactivity Index, which measures perspective taking, empathic concern, personal distress, and fantasy with four individual subscales, utilising a five-point Likert-type response scale with the anchors 0 = *Does not describe me well* and 4 = *Describes me very well*. Measures are presented below with the dependent variables (grief and loss reactions, closure and gratitude) presented first, followed by the independent variables. The full items that make up newly devised scales can be obtained by contacting the study author.

#### Grief and loss reactions

Feelings of grief and loss were measured with new and existing measures. First, a series of new items were developed based on examination of general grief and loss literature (e.g., [105–110]), as well as examination of studies of grief following the exit of a TV character from a series (e.g., [89, 92]), or the death of a celebrity (e.g., [94]). Based on parallel and factor analysis on these items, two new scales were devised: Grief Emotions (8 items; possible score range = 8–40; e.g., “I am angry that *Neighbours* has ended”, “I miss *Neighbours*”) and Non-acceptance (5 items; possible score range = 5–25; e.g., “I am still in denial that *Neighbours* has ended”). Higher scores indicate greater grief and loss emotions (Grief Emotions) and lesser acceptance (Non-acceptance) of the end of the series.

Second, the existing 13-item Parasocial Breakup measure [42] was used. The Parasocial Breakup measure examines feelings and behaviours associated with a favourite character no longer being on the air. It was chosen for inclusion given it taps into loss reactions and distress similar to those experienced at the end of a real-world relationship. The measure was originally used to measure fans’ reactions to the end of the TV series *Friends*; for the present study *Neighbours* was substituted into items. An example item is, “Now that my favorite *Neighbours* character is off the air, I feel more lonely”. Scores can range from 13–65, with higher scores indicating greater feelings of parasocial breakup and distress. Reliability (Cronbach’s alpha) was reported previously as .81 [42].

#### Closure related to the finale and gratitude for the series

To measure closure at the end of the series and gratitude for the series, new items were developed based on previous literature on gratitude, particularly in the context of loss (e.g., [111–113]), as well as items written to examine rituals enacted by participants to say goodbye to the series. Based on parallel and factor analysis, two scales were devised: Closure (7 items; possible score range = 7–35; e.g., “The final episode provided closure for me”) and Gratitude (3 items; possible score range = 3–15; e.g., “I am glad that *Neighbours* was in my life”). Higher scores indicate greater perceptions of closure and ability to celebrate the series, and greater gratitude for the series having been in one’s life, respectively.

#### Motives for watching

Motives for watching the series were measured using an adapted version of the Local Television News Viewing Motives scale [19], which examines reasons for watching television news with three scales: Exciting Entertainment, the extent to which the viewer finds a program entertaining and exciting; Pass Time, the extent to which the viewer watches the program to occupy or pass time; and Information, watching the program to learn about oneself, to learn new skills, and to have something to speak about with others. Item wordings were slightly modified, one Information item was removed as it applied to news but not drama series viewing, and additional items were written to further examine learning through viewing and connection with others (reflected in the original Information scale) and, of specific interest to the present study, exposure to different experiences or lifestyles through storylines and characters. Participants were prompted to consider reasons for watching the series with the beginning of the sentence being “I Watched *Neighbours*” followed by the reason (e.g., “Because it exposed me to different lifestyles”).

Based on parallel and factor analysis, four scales were devised. Two scales replicated the Exciting Entertainment (5 items; possible score range = 5–25; e.g., “Because it was enjoyable”) and Pass Time (5 items; possible score range = 5–25; e.g., “Because it gave me something to occupy my time”) original scales. The two new scales were: Experiences (7 items, 5 new and 2 Information items; possible score range = 7–35; e.g., “So I could learn about things that I haven’t personally experienced”) and Feeling Part of a Community (4 items, 3 new and 1 Information item; possible score range = 4–20; e.g., “So I could talk with other people about what had happened in the show”). Higher scores on each scale indicate greater use of the series for the particular motive.

#### Degree of being a fan of the series

Self-identification as a fan and perceiving that one was part of the fan community were measured with new items (for self-identification) and adapted items from a previous study (for being a part of the fan community) [33]. Based on parallel and factor analysis, two scales were devised: Degree of Being a Fan (5 items; possible score range = 5–25; e.g., “I am a big fan of *Neighbours*”) and Being Part of the Fandom (4 items; possible score range = 4–20; e.g., “I see myself as belonging to the *Neighbours* fanbase”). Higher scores indicate greater self-identification as a fan of the series and greater connection to the fandom, respectively.

#### Parasocial relationships and empathy

Participants were asked to think about their favourite character and then complete items about their parasocial relationship with and empathy toward that character. Parasocial relationships were measured using items from the Parasocial Interaction Scale [19], which measures positive emotions (e.g., liking), thoughts (e.g., perceptions of belonging and closeness), and behaviours (e.g., comparing ideas) towards a favourite parasocial figure. Items were modified to reflect a drama series and its characters, with some items removed that were not applicable to a fictional drama series. Empathy towards one’s favourite character was measured using new items based on research into perspective taking and emotional empathy (e.g., [56, 57, 59, 114]). Items examined the taking of the perspective of a favourite character, similar emotional responses to the character (identification or parallel emotions), and feelings of care and concern for them.

Based on parallel and factor analysis, initially two factors, one each of parasocial interaction and empathy, were calculated. However, these factors were very highly correlated (*r =* .86, *p <* .001). The decision was made to investigate whether both sets of items might instead be combined into one measure. This was decided based on a several considerations, including some previous work having included the concept of empathy within definitions of the parasocial relationship concept, and other studies having similarly found a strong relationship between parasocial relationship and empathy measures (e.g., [49, 83]). From a statistical perspective, such highly correlated separate variables would not be able to be used in subsequent analyses suggesting the need to amalgamate items [103]. With these considerations in mind, a one-factor solution was subsequently specified in factor analysis. All items loaded on this factor and, therefore, a single-factor Parasocial Empathic Interaction scale consisting of 26 items was devised. Example items include, “When my favourite character reveals how they feel about a particular issue, it helps me make up my own mind about that issue” (parasocial interaction item) and “I imagine how I would feel if I were in my favourite character’s situation” (empathy item). Scores can range from 26–130, with higher scores indicating greater parasocial (e.g., liking) and empathic interaction (e.g., taking a character’s perspective and experiencing similar feelings to the character or an emotional response to their situation) with a favourite character.

#### Perspective taking, empathic concern, personal distress, and fantasy

Tendency to engage in perspective taking and to experience empathic concern, personal distress, and fantasy were measured using the Interpersonal Reactivity Index [76], which consists of four seven-item subscales measuring four components of empathy: Perspective-Taking, the tendency to take the perspectives of others (e.g., “When I’m upset at someone, I usually try to ‘put myself in their shoes’ for a while”); Empathic Concern, the tendency to experience care and concern for others (e.g., “I often have tender, concerned feelings for people less fortunate than me.”); Personal Distress, the tendency to experience distress in difficult interpersonal situations (e.g., “When I see someone who badly needs help in an emergency, I go to pieces”); and Fantasy, the tendency to engage cognitively and emotionally with fictional characters in various mediums (e.g., “I really get involved with the feelings of the characters in television series, novels, or movies”). Total scores for each subscale can range from 0–28, with higher scores indicating greater disposition to experience the particular empathy component. Reliability was reported > .70 for each scale in initial validation [76].

### Procedure

The survey was open from 31 July to 10 November 2022 (Australian time zones) and was hosted on the Qualtrics platform. After reading an Information Sheet and indicating their voluntary consent to participate, participants completed demographic items and items related to viewing habits, followed by items measuring degree that they were a fan of the series, reasons for watching, parasocial interaction, parasocial empathy, parasocial breakup, grief, and closure and gratitude following the end of the series. The study was approved by CQUniversity Human Research Ethics Committee (Application ID 0000023677).

### Data analysis

Data was analysed using IBM SPSS Statistics (Version 27). Prior to data analysis, cases were deleted if they had completed no or minimal items (e.g., a few items). Following this, cases were removed if they missed entire scales or, on visual inspection of the data, had responded frivolously (e.g., selecting 5 for every item in the survey). A small number of further case deletions were undertaken if participant criteria were not met. There were minimal missing values on individual items, with these replaced by a series mean [102, 103]. Parallel and factor analysis was conducted so that totals for all scales could be calculated and, following this, examination of multivariate and univariate outliers was undertaken. This resulted in the removal of 37 multivariate outliers (using Mahalanobis distance, *p <* .001) [115]. The final sample for analysis consisted of 1289 participants. Nineteen univariate outliers (-3.29 *< z*s *>* 3.29) were identified with the relevant individual scale scores deleted, but the rest of the participants’ data retained. While there was some skewness and kurtosis on certain variables, this was not excessive [103], and so no data was transformed but metrics indicating appropriateness of data for analysis were examined during regression analyses.

Following data cleaning, descriptive and correlational analyses were undertaken. Multivariate multiple regression was then conducted to investigate predictors of the multiple dependent variables, followed by each individual dependent variable regressed on independent variables (following procedures in [116]).

## Results

### Descriptive statistics

Table 2 presents mean scores, standard deviations, Cronbach’s alphas (*α*), and (for ease of interpretation of participant responses) possible score ranges for each measure.

**Table 2:** Means and standard deviations for measures.

Scale	*M* (SD)	Cronbach’s *α*	Possible score range
Dependent variables			
Grief Emotions	33.72 (5.39)	.88	8–40
Non-acceptance	17.65 (4.43)	.82	5–25
Parasocial Breakup	44.95 (9.08)	.89	13–65
Closure	23.85 (5.26)	.82	7–35
Gratitude	13.84 (1.28)	.65	3–15
Independent variables			
Motives–Exciting Entertainment	21.58 (2.34)	.72	5–25
Motives–Pass Time	9.69 (4.60)	.89	5–25
Motives–Experiences	25.24 (5.76)	.91	7–35
Motives–Community	14.43 (3.32)	.77	4–20
Degree of Being a Fan	22.45 (2.54)	.77	5–25
Being Part of the Fandom	14.58 (2.96)	.70	4–20
Parasocial Empathic Interaction	90.23 (16.92)	.95	26–130
Perspective-Taking	18.35 (4.51)	.74	0–28
Empathic Concern	21.03 (4.24)	.69	0–28
Personal Distress	13.00 (5.29)	.78	0–28
Fantasy	16.23 (5.54)	.74	0–28

Participants reported a high level of grief emotions associated with the end of the series, and they had difficulty accepting that it had ended. Participants reported lesser closure related to the end of the series. However, they did report being very grateful for the series.

Mean scores for viewing motives indicated that participants watched the series because they found it entertaining and exciting, somewhat to experience other lifestyles/ideas and to discuss the show with others, but not to merely pass time. Overall, participants were devoted fans. They self-identified as being very big fans of the series and felt fairly connected to the *Neighbours* fandom. Participants reported that they experienced a parasocial empathic relationship with their favourite character to a fairly high extent. A total of 72 individual characters were chosen by participants as their favourite character. Most were current characters, but there were some characters that had left the series before the finale, as well as older characters going back to the 1980s. The most frequently selected characters were Susan Kennedy, Toadie Rebecchi, Paul Robinson, Chloe Brennan, Terese Willis, and Karl Kennedy.

For empathic tendencies, participants reported higher levels of perspective taking and empathic concern, with more moderate levels of tendency to fantasy and relatively low levels of personal distress.

Pearson’s correlation coefficients (*r*) are reported in Table 3. Grief and loss measures were moderately to strongly correlated, with measures of grief emotions, non-acceptance, parasocial breakup, and gratitude positively correlated with one another. All grief and loss measures except for gratitude were negatively correlated with closure.

**Table 3:** Correlation coefficients between variables.

	Emot	Acc	Break	Clos	Grat	Gen	Age	Ent	Pass	Exp	Comm	Fan	Fand	PEI	PT	EC	PD
Acc	.66***																
Break	.66***	.52***															
Clos	-.28***	-.37***	-.17***														
Grat	.42***	.27***	.44***	.14***													
Gen	.01	.08**	-.06*	-.13***	-.13***												
Age	-.11***	-.14***	-.22***	-.15***	-.23***	.15***											
Ent	.45***	.39***	.43***	-.09**	.27***	.03	-.17***										
Pass	-.03	-.02	.12***	.12***	-.04	-.03	-.20***	-.03									
Exp	.42***	.39***	.50***	-.03	.27***	.03	-.18***	.48***	.17***								
Comm	.41***	.37***	.51***	.01	.33***	-.02	-.14***	.42***	.19***	.62***							
Fan	.52***	.40***	.48***	-.07*	.42***	.004	-.12***	.45***	-.07*	.37***	.37***						
Fand	.47***	.38***	.50***	-.04	.32***	.01	-12***	.51***	-.02	.46***	.57***	.52***					
PEI	.49***	.41***	.71***	-.003	.37***	-.01	-.20***	.49***	.16***	.67***	.63***	.44***	.53***				
PT	.03	.05	.10***	.05	.13***	.09**	.02	.11***	-.01	.13***	.12***	.14***	.11***	.13***			
EC	.12***	.11***	.16***	-.04	.21***	.11***	.01	.15***	-.08**	.13***	.11***	.19***	.13***	.16***	.52***		
PD	.12***	.09**	.19***	-.01	.03	.05	-.08**	.03	.14***	.18***	.12***	.03	.08**	.24***	-.11***	-.01	
FS	.28***	.25***	.39***	.02	.29***	.06*	-.21***	.24***	.08**	.36***	.32***	.28***	.30***	.50***	.23***	.35***	.25***

Note

*** *p <* .001

** *p <* .01

* *p <* .05. Emot = Grief Emotions; Acc = Non-acceptance; Break = Parasocial Breakup; Clos = Closure; Grat = Gratitude; Gen = Gender (0 = Not female, 1 = Female); Age = Age in Years; Ent = Motives–Exciting Entertainment; Pass = Motives–Pass Time; Exp = Motives–Experiences; Comm = Motives–Community; Fan = Degree of Being a Fan; Fand = Being Part of the Fandom; PEI = Parasocial Empathic Interaction; PT = Perspective-Taking; EC = Empathic Concern; PD = Personal Distress; FS = Fantasy.

### Predicting feelings of grief and loss

Four dependent variables measuring grief and loss experiences were included in analyses to test the main hypotheses. These were (1) grief emotions; (2) not accepting the end of the series; (3) parasocial breakup; and (4) closure. Gratitude was not included as there was very little variability in scores (i.e., most fans were very grateful for the series), meaning that the measure was not suitable for regression analysis [103].

Multivariate multiple regression analysis revealed that the overall model with the four dependent variables was significant, *Pillai’s trace =* .79, *F*(52, 5032) = 23.95, *p <* .001. The dependent variables were then regressed on the independent variables with four separate regressions run. Independent variables entered in each of the four regressions were (1) gender, coded as 1 = female or 0 = not female (so that all participants could be included); (2) age; (3) the four motives for watching the series (exciting entertainment, pass time, experiences, community); (4) degree of being a fan and fandom; (5) parasocial empathic interaction with a favourite character; and (6) the four empathic tendency measures (perspective taking, empathic concern, personal distress, and fantasy). Assumptions for regression were assessed [102, 103], including those related to multicollinearity and independent errors, since some of the variables were quite highly correlated. These were found to all be within acceptable ranges, suggesting the suitability of the data for analysis.

#### Grief emotions

For Grief Emotions (Table 4), variables explained 39% of population variance, *F*(13, 1258) = 61.68, *p <* .001. Greater grief emotions were associated with greater use of the series for exciting entertainment (*β =* .13) and experiences (*β =* .07), greater self-identification as a fan (*β =* .28) and feeling a part of the fandom (*β =* .11), and greater parasocial empathic interaction with a favourite character (*β =* .16). Lesser grief emotions were associated with greater use of watching the series to pass the time (*β =* -.06), and greater perspective taking (*β = —*.09).

**Table 4:** Multiple regression for predicting grief emotions.

	B	95% CI Lower	95% CI Upper	*SE*_*B*_	*β*	*P*
Gender (Female)	0.10	-0.46	0.65	0.28	0.01	.74
Age	0.000	-0.02	0.02	0.01	-0.001	.97
Motives–Exciting Entertainment	0.30	0.17	0.43	0.06	0.13	< .001
Motives–Pass Time	-0.07	-0.12	-0.01	0.03	-0.06	.02
Motives–Experiences	0.07	0.01	0.13	0.03	0.07	.03
Motives–Community	0.07	-0.03	0.17	0.05	0.04	.17
Degree of Being a Fan	0.59	0.48	0.70	0.06	0.28	< .001
Being Part of the Fandom	0.20	0.09	0.31	0.06	0.11	< .001
Parasocial Empathic Interaction	0.05	0.03	0.07	0.01	0.16	< .001
Perspective-Taking	-0.11	-0.17	-0.05	0.03	-0.09	< .001
Empathic Concern	0.03	-0.03	0.10	0.03	0.03	.33
Personal Distress	0.04	-0.01	0.08	0.02	0.04	.12
Fantasy	0.03	-0.02	0.08	0.03	0.03	.27

#### Not accepting end of the series

For Non-acceptance of the End of the Series (Table 5), variables explained 28% of population variance, *F*(13, 1262) = 37.21, *p <* .001. Lesser acceptance of the end of the series was associated with being female (*β =* .09), greater use of the series for exciting entertainment (*β* = .12), experiences (*β* = .11), and community (*β* = .08), greater self-identification as a fan (*β* = .18), and greater parasocial empathic interaction with a favourite character (*β* = .09). Greater acceptance was associated with older age (*β* = -.05) and greater use of the series to pass the time (*β* = -.07).

**Table 5:** Multiple regression for predicting not accepting ending of series.

	B	95% CI Lower	95% CI Upper	*SE*_*B*_	*β*	*P*
Gender (Female)	0.88	0.38	1.38	0.25	0.09	< .001
Age	-0.02	-0.04	-0.001	0.01	-0.05	.04
Motives–Exciting Entertainment	0.23	0.12	0.35	0.06	0.12	< .001
Motives–Pass Time	-0.06	-0.11	-0.01	0.03	-0.07	.01
Motives–Experiences	0.08	0.03	0.13	0.03	0.11	.002
Motives–Community	0.11	0.02	0.20	0.05	0.08	.02
Degree of Being a Fan	0.32	0.22	0.42	0.05	0.18	< .001
Being Part of the Fandom	0.10	0.000	0.20	0.05	0.07	.05
Parasocial Empathic Interaction	0.02	0.004	0.04	0.01	0.09	.02
Perspective-Taking	-0.05	-0.11	0.001	0.03	-0.05	.06
Empathic Concern	0.01	-0.05	0.07	0.03	0.01	.68
Personal Distress	0.01	-0.04	0.05	0.02	0.01	.80
Fantasy	0.03	-0.02	0.08	0.02	0.04	.23

#### Parasocial breakup

For Parasocial Breakup (Table 6), variables explained 55% of population variance, *F*(13, 1262) = 117.34. Greater parasocial breakup distress was associated with greater self-identification as a fan (*β* = .17) and feeling a part of the fandom (*β* = .08), greater parasocial empathic interaction with a favourite character (*β* = .52), and greater personal distress (*β* = .04). Lesser distress was associated with being female (*β* = -.05) and older age (*β* = -.06).

**Table 6:** Multiple regression for predicting parasocial breakup.

	B	95% CI Lower	95% CI Upper	*SE*_*B*_	*β*	*P*
Gender (Female)	-0.98	-1.79	-0.17	0.41	-0.05	.02
Age	-0.05	-0.09	-0.02	0.02	-0.06	.002
Motives–Exciting Entertainment	0.12	-0.06	0.31	0.09	0.03	.20
Motives–Pass Time	0.05	-0.03	0.12	0.04	0.02	.26
Motives–Experiences	-0.04	-0.12	0.05	0.04	-0.03	.37
Motives–Community	0.14	-0.01	0.29	0.08	0.05	.07
Degree of Being a Fan	0.61	0.44	0.77	0.08	0.17	< .001
Being Part of the Fandom	0.24	0.08	0.39	0.08	0.08	.004
Parasocial Empathic Interaction	0.28	0.25	0.31	0.02	0.52	< .001
Perspective-Taking	-0.03	-0.12	0.06	0.05	-0.02	.47
Empathic Concern	0.07	-0.03	0.17	0.05	0.03	.14
Personal Distress	0.07	0.01	0.14	0.04	0.04	.04
Fantasy	0.02	-0.06	0.10	0.04	0.01	.61

#### Closure

For Closure (Table 7), variables explained 7% of population variance, *F*(13, 1262) = 6.75, *p <* .001. Greater closure was associated with greater use of the series to pass the time (*β* = .08) and greater perspective taking (*β* = .10). Lesser closure was associated with being female (*β* = -.11), older age (*β* = -.14), and greater use of the series for exciting entertainment (*β* = -.10).

**Table 7:** Multiple regression for predicting closure.

	B	95% CI Lower	95% CI Upper	*SE*_*B*_	*β*	*P*
Gender (Female)	-1.32	-1.99	-0.64	0.35	-0.11	< .001
Age	-0.07	-0.10	-0.04	0.01	-0.14	< .001
Motives–Exciting Entertainment	-0.23	-0.39	0.08	0.08	-0.10	.003
Motives–Pass Time	0.09	0.02	0.15	0.03	0.08	.01
Motives–Experiences	-0.04	-0.11	0.03	0.04	-0.04	.29
Motives–Community	0.06	-0.07	0.18	0.06	0.03	.39
Degree of Being a Fan	-0.08	-0.22	0.06	0.07	-0.04	.27
Being Part of the Fandom	-0.01	-0.14	0.13	0.07	-0.003	.93
Parasocial Empathic Interaction	0.01	-0.02	0.04	0.01	0.03	.52
Perspective-Taking	0.12	0.05	0.19	0.04	0.10	.001
Empathic Concern	-0.07	-0.15	0.02	0.04	-0.05	.11
Personal Distress	-0.01	-0.07	0.04	0.03	-0.01	.62
Fantasy	0.01	-0.05	0.08	0.03	0.01	.70

## Discussion

The purpose of this study was to investigate what factors influenced fans’ grief and loss reactions to the end of the television series *Neighbours*. Grief and loss experiences were greater in viewers who used the series for entertainment, exposure to lifestyles and experiences, and community, but not to pass time; self-identified as being big fans of the series and felt a part of the fandom; and reported having stronger parasocial empathic relationships with characters. Lesser closure was associated with being female, older, and using the series for entertainment to a greater extent and lesser use of it to pass the time. Findings extend previous work regarding the importance of parasocial relationships, considering parasocial grief in the context of a long-running narrative series.

Grief reactions from fans involved significant emotions, cognitions (largely disbelief), and perceptions of loss. Fans seemed to be experiencing quite strong grief, reflecting the importance of the series and relationships with characters to their lives and a sense of parasocial breakup or loss [42]. The strength of these reactions, such as anger and sadness, lesser acceptance and lesser closure suggest that fans were at an earlier part of processing their grief reactions (e.g., see [17, 18]), which makes sense given the time the survey was distributed. Despite this, fans did indicate that they were grateful that the series had been in their lives. This may reflect that, while the end of the series was shocking to fans (e.g., [9, 10]), they did have time between the announcement in March and the screening of the final episode in July 2022 to reflect on the series, celebrate and commemorate it, and take stock of what the series had given them.

Motives for watching the series were focused on entertainment, including aspects of excitement or thrill, with some use of the series to experience lifestyles and situations different to one’s own or to discuss the show with others, but not as a way to pass time. These findings reflect the use of fictional series to meet both hedonic and eudaimonic motives [20, 23, 26, 28], involving pleasure, self-reflection, reflection on issues, and meeting of wellbeing needs. That all investigated viewing motive factors except passing the time were associated with greater grief and loss reactions suggests that participants were engaging in active rather than passive engagement with the series (see [117]). Perhaps this also helps explain the degree of grief felt in the present study given the likely greater difficulty in meeting such needs with a new series versus finding a series to merely pass the time.

The degree of engagement is likely related to the viewing habits and history of the sample. The mean age of participants was only a few years older than the series itself suggesting that, for many fans, the series had been in their lives for decades and/or since they were children. Most participants, however, watched the series alone or reported a mixture of watching it alone and viewing with others. This suggests choice to watch the series and that participants were not doing so solely because other members of their household watched the show, or because the show was background viewing. Participants did feel somewhat connected to the fan community and did connect with others to discuss the series. Fan identity and perceptions of belonging to a fandom are associated with greater wellbeing [34], although work in this area is needed [35, 36]. Similarly, understanding by others (e.g., friends) of one’s parasocial relationships is associated with the strength of the parasocial bond [118]. Therefore, it is not surprisingly that feeling a part of the fan community and considering oneself a devoted fan were predictors of greater grief reactions following the series’ end.

Parasocial interaction involving empathic engagement with a favourite character was a consistent predictor of grief and loss reactions. This supports research with other series, where stronger parasocial relationships are associated with greater perceptions of a loss akin to a breakup when the series ends (e.g., [44, 45, 93]). The present study extends previous findings by more directly considering different grief reactions and perceptions of closure and gratitude. Series that screen for many years afford a unique opportunity for fans to develop long-standing bonds with characters and storylines to the extent that they engage with the series over time [119, 120]. Parasocial relationships are likely cyclical with viewing habits, with stronger parasocial relationships associated with continued viewing, and such engagement in turn likely further strengthening these relationships [51].

Both perceptions of parasocial relationships with favourite characters and empathy towards these characters were high. Like previous studies (e.g., [49, 83]), items measuring parasocial relationship strength and character empathy were highly correlated. The decision was made, therefore, to amalgamate items. This high correlation is to be expected for several reasons. Empathy strengthens bonds between individuals and allows understanding [60]. In the case of fictional narratives, *mutual* understanding (i.e., between empathiser and recipient of empathy) is not possible [41]. However, perceptions of understanding one’s favourite character and empathising with them are strongly associated with perceptions of a stronger parasocial relationship [53]. Previous studies have found that greater parasocial interaction (relationship) with a character is associated with beliefs that one understands and can predict the characters’ thoughts, feelings, and behaviours [29]. Prediction is an important outcome of empathy [57, 70, 121], and so (presumed) understanding likely fosters closeness with and liking of characters through a reduction in uncertainty regarding a character’s perspective and feelings [29].

The relationship between parasocial interaction and empathy has been examined in several studies, but there is ambiguity as to the nature of how parasocial relationships depend on empathy and how the relationship results in empathy for a character [68, 71]. This is perhaps reflected in measures of parasocial relationships (and viewing motives) sometimes including items measuring aspects associated with empathy such as perceived similarity, interaction, liking, vicarious emotional release, and feelings of belonging (see [20]), and even enjoyment of character’s actions [122]. There is contestation within the wider empathy research [54, 55, 57, 70] as to what extent these components are or are not empathy. However, the present findings reflect the potential utility of considering the relationships formed with characters in terms of the *parasocial interaction* (e.g., bonding, liking) and the empathic *processes* (e.g., perspective taking) and *outcomes* (e.g., emotional empathy, behavioural reactions) that are implicated in the fan-character relationship. What is needed is more exploration regarding how best to differentiate these and other factors or to conceptualise them within larger models (e.g., [123]) that consider the maintenance and intensification of the parasocial bond.

While not a central concern of the present study, some participants interestingly chose to reflect on characters who were no longer in the series. Whether this reflects the nature of television viewing now, with the opportunity to watch old episodes allowing the viewer to form (or re-form) relationships with former characters, or that participants who had watched for many years still felt that had a relationship with previous characters is unclear. If the former, this does suggest the need for research to consider viewing habits, particularly asynchronous viewing [124], and the ability to continue to have relationships with a concluded series [125]. If the latter, this adds additional complexity to ideas regarding how parasocial interactions become relationships [39], and the extent to which they are assimilated into one’s memory even after the character is no longer present. It is also important to note that while asking about one favourite character is common practice in research [41] and allows a measure of the strongest parasocial relationship, it is likely that in the case of series with large casts, parasocial relationships are formed with several characters.

Empathic tendencies were not particularly associated with grief reactions in regression analysis, suggesting that propensity to engage in empathy, particularly with fictional characters, may not be as proximally related to grief responses when both empathic tendency and specific empathy for characters are measured. Instead, it may be that degree of engagement with characters is more important to understanding grief reactions related to these characters [126–128]. Of the four trait measures, perspective taking was associated in regression analysis with lesser grief reactions and greater perceptions of closure. This may appear surprising since empathy for a favourite character did predict grief and loss experiences. However, previous studies have found that while related, empathic tendencies are more moderately associated with engagement with fictional characters [129]. In addition, perspective taking implies a move between a self- and other perspective [56, 60], which may highlight to viewers the fictional nature of the engagement and result in lesser grief and loss reactions.

Since the study participants were very committed fans, variables such as years watching or number of episodes watched per week were not included in regression analyses due to limited variability in responses. However, two demographic variables, gender (being female) and (older) age did predict lesser acceptance (for females) of the end of the series and closure (for females and older participants). Previous studies have found greater parasocial relationships in both groups [34, 130]. Age is likely implicated in years watching and, therefore, there may be more difficulty in feeling closure. Female gender and grief reactions might be tied into aspects such as forming stronger relationships with parasocial characters, or other factors such as higher self-reported empathy in females (e.g., [131]). However, female and older participants also reported lesser parasocial breakup distress. This suggests that there may be more complex relationships underlying these results where, because the loss is unacknowledged or relationships are not seen as having ended, breakup distress or active grieving does not occur. While intriguing, this requires further investigation.

### Strengths and limitations

The present study collected timely information on fans’ interactions with an increasingly scarce program in media: the long-running, continuing storyline show. The sample was large, and data was collected very soon after the end of the series. Viewing habits and connection to the series and its characters were considered in line with previous work, extending understanding to a direct examination of grief and loss at the end of important and long-running (sometimes generational) parasocial relationships [132].

However, the study findings need to be considered based on methodological design. Like most examinations of parasocial relationships, an exploratory cross-sectional approach was chosen. This means that while associations were found, the nature of these relationships from a causation perspective cannot be determined.

Grief and loss measures were developed based on existing literature, but were new, and so further examination of these and the concepts they purport to measure should be undertaken. Measures were also often highly correlated. It could be argued that perhaps a more confined set of variables should have been investigated. However, findings including high correlations between concepts reflect previous studies and the multifaceted nature of parasocial relationships.

The sample was by its very nature self-selecting with participants often big fans of the series. Other viewers were excluded from participation such as those sad at the demise of a series they once watched and for whom there is likely associated nostalgia. Attempts were made to recruit a diverse sample by targeting large social media groups where presumably there is variability in interest in the series, but the need to collect data shortly after the final episode did mean that explicit attempts to recruit for less devoted fans were not undertaken.

Finally, the cross-sectional nature of the study means that we do not know how grief and loss change over time. In reflecting on engagement with the series soon after it had ended, participants may have overestimated their degree of connection with the series and characters. A study that measures reactions over time in the same set of participants would provide further insight into the course of grief in fans. Participants were given the opportunity to reflect further on the end of the series in response to a one item open-ended question; this data will be reported in a future paper.

### Implications

This study builds on theory and research into viewer engagement with television series and fictional media in several ways. The study adds to the small literature base on how fans process the loss of their favourite series and characters, providing data that can support conceptualising this as a grief similar to other loss reactions. The study is, therefore, an initial step in theorising grief reactions in fan communities. Further investigation into the ways in which parasocial relationship dissolution is similar and qualitatively different from other relationship endings could be particularly fruitful in developing a larger theory regarding parasocial loss and grief.

The study investigated what might influence the nature of fans’ grief, finding that when empathy for characters is measured, it shares much commonality with parasocial relationship measurement. This should be further investigated. Important will be examining empathy and parasocial interaction in theoretical models with variables that have been found to shape the parasocial relationship [48, 130], those that are common to a person’s real and parasocial relationships, and those that speak to how a person see themselves and others (e.g., perceptions of closeness [133] and motivation to engage in relationships [134]). Further investigation of different modes of parasocial and empathic relationships, such as connections with film, television, live performance, and book characters is needed, so that a wider theory can be developed.

Finally, the findings have implications for those working with anyone who is experiencing such a loss and for whom the loss is particularly acute and upsetting. Based on the findings, examining reasons for watching, nature of parasocial and real-world interactions, and how identified needs in clients could be met by other parasocial relationships or, if excessive, redirected to be met in the real world would be useful.

## Conclusion

This study found that fans may experience significant feelings of grief at the end of a series, with fan reactions influenced by the place of the series in their lives and the relationships they have formed with characters. While different to real-world social interactions, these parasocial relationships are taken seriously by fans and are important to them. In this way, they should be honoured with increased research attention.

As a postscript, in November 2022, after data collection had ceased, it was announced that the series would be revived in late 2023 [135]. The series relaunched in September 2023 [136]. While fans were very satisfied with this outcome and fan reactions to the end of the series (and finale ratings) likely played a part, there was no indication at the time of the screening of the finale and data collection that this would occur. Therefore, the reactions collected in this study of a once-in-a-generation end to a series were unique and not likely to be repeated in the literature for some time. Future examination of fans’ reacquainting with the series, including examinations of their joy and satisfaction with the continuation of the story and their bonds with returning and new characters, is warranted. Researchers and audiences alike should stay tuned for the next episode of *Neighbours*.

## Supporting information

S1 Checklist *PLOS ONE* clinical studies checklist. (DOCX)

S2 Checklist STROBE statement—Checklist of items that should be included in reports of observational studies. (DOCX)
